# Duodenal nodular lymphoid hyperplasia in a patient with IgA deficiency

**DOI:** 10.1002/ccr3.3298

**Published:** 2020-09-08

**Authors:** Hanae Ida, Dai Maruyama, Akiko Miyagi Maeshima, Takahiro Kamiya, Tomohiro Morio, Koji Izutsu

**Affiliations:** ^1^ Department of Hematology National Cancer Center Hospital Tokyo Japan; ^2^ Department of Pathology National Cancer Center Hospital Tokyo Japan; ^3^ Department of Pediatrics and Developmental Biology Tokyo Medical and Dental University Tokyo Japan

**Keywords:** Common variable immunodeficiency, Esophagogastroduodenoscopy, IgA deficiency, Lymphoma, Nodular lymphoid hyperplasia

## Abstract

Most patients with IgA deficiency are asymptomatic, but duodenal nodular lymphoid hyperplasia is one symptom known to be associated with common variable immunodeficiency (CVID), including selective IgA deficiency and agammaglobulinemia.

## Graphical abstract

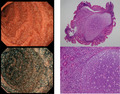

## 1 INTRODUCTION

**Figure 1:**
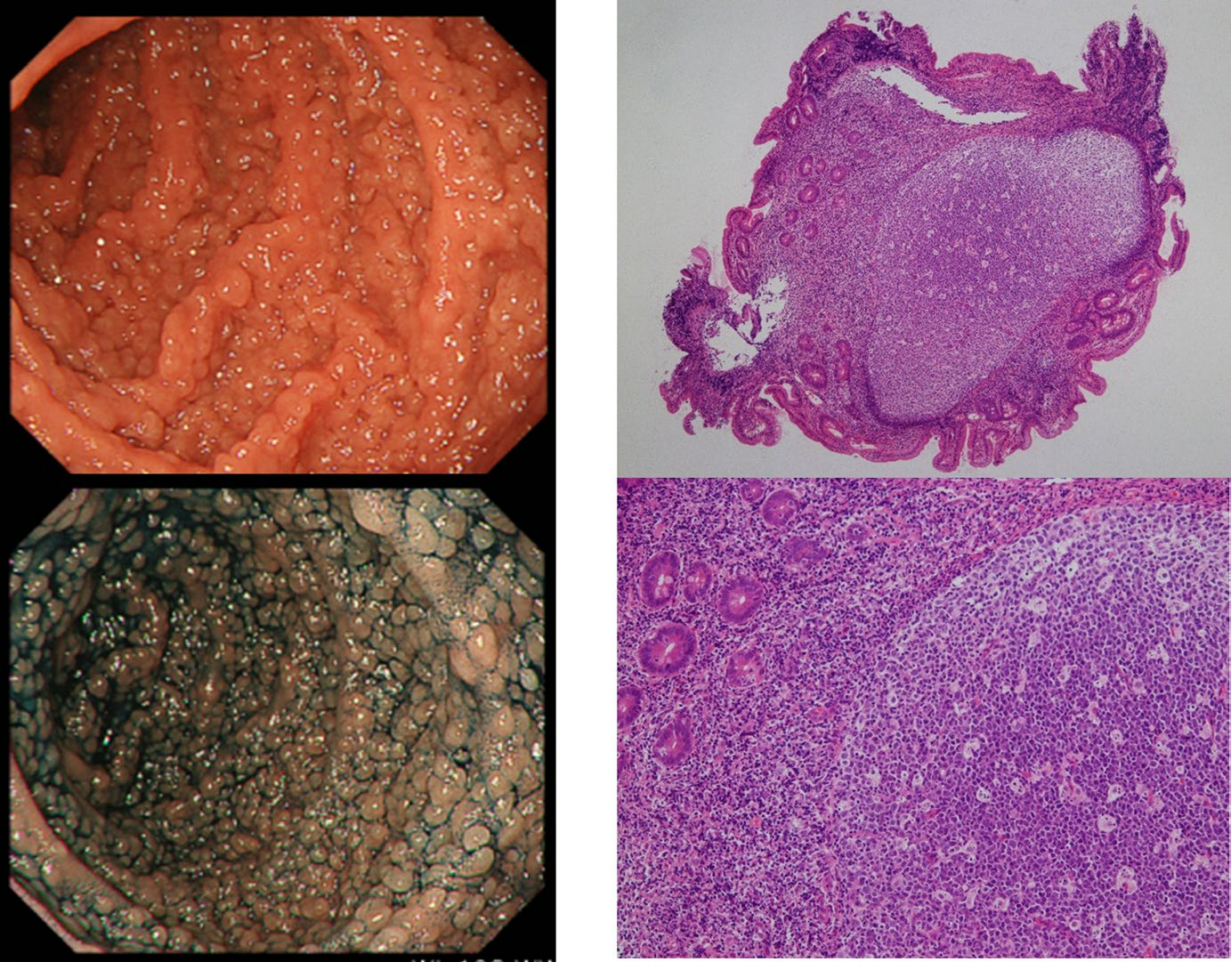
Left, Endoscopic image showing multiple small nodules. There were many nodules of 5 mm or less in the entire duodenum, which were accompanied by white and dilated blood vessels (so‐called multiple lymphoid polyposis). The findings reflected a differential diagnosis of follicular lymphoma and mantle cell lymphoma, but the lesions had no white enlarged villi and were relatively the same size. Hyperplasia of reactive lymphoid follicles was also a possibility. Right, Reactive hyperplasic, mitotically active germinal centers with well‐defined lymphocyte mantles were observed

A 29‐year‐old woman with no past medical history presented with vomiting after eating. Esophagogastroduodenoscopy (EGD) revealed multiple nodular lymphoid hyperplasia (NLH) of around 5 mm in size in the duodenum (Figure 1; left). Malignant lymphoma was suspected by the local doctor, and she was referred to our hospital. Pathological examination of the biopsy specimen of NLH revealed reactive hyperplasic, mitotically active germinal centers with well‐defined lymphocyte mantles, morphological, and immunohistochemistry findings were not consistent with malignant lymphoma (Figure 1, right). Flow cytometry analysis of the biopsy specimen also demonstrated no evidence of monoclonal lymphoid cells. Regarding serum immunoglobulin levels, IgA was undetectable, IgM was at the lower limit of normal, and IgG was within normal limits. No M‐protein was detected by serum and urine protein immunofixation electrophoresis; and imbalance of serum‐free light chains was not observed. Duodenal polyposis was diagnosed as being associated with IgA deficiency, IgM hypogammaglobulinemia and specific antibody deficiency, with a decrease in CD27‐positive B cells.

Most patients with IgA deficiency are asymptomatic, but duodenal NLH is a symptom known to be associated with common variable immunodeficiency (CVID), including selective IgA deficiency and agammaglobulinemia.^1^, ^2^ When such EGD and histological findings are observed, the possibility of IgA deficiency should be considered.

## CONFLICT OF INTEREST

None declared.

## AUTHOR CONTRIBUTIONS

HI and DM: managed and diagnosed the patient and were responsible for collection and interpretation of the data and wrote the manuscript. AMM: made pathological diagnosis of the patient. TK and TM: managed and diagnosed the patient. KI: interpreted the data. All reviewed and approved the final version of the manuscript.

## ETHICAL APPROVAL

No ethical approval is required for case reports in our institution.
